# Scaling up World Organization of Family Doctors membership within the African region

**DOI:** 10.4102/phcfm.v17i1.5072

**Published:** 2025-09-15

**Authors:** Jane F. Namatovu

**Affiliations:** 1 Department of Family Medicine, School of Medicine, Makerere University College of Health Sciences, Kampala, Uganda

## Context

In Africa, family medicine training was started in the 1960s in South Africa and Nigeria. Since then, new training programmes continue to be started within and among African countries. As new programmes are developed, the number of family physicians has also progressively increased. This called for African family physicians to link with their colleagues in other parts of the world to learn together, share experiences, network and collaborate. The World Organization of Family Doctors (WONCA) provided a platform to address this new need.

World Organization of Family Doctors started in 1972 in Australia and currently has seven regions around the world. The regions include Africa, Eastern Mediterranean, Asia Pacific, South Asia, Europe, North America and Ibero-Americana. Among the regions, Africa has the highest number of 54 countries and appears to have more cultural and linguistic diversity given the colonial histories. English, French and Portuguese are the main national languages in the region. Most countries are English speaking with a few Portuguese-speaking. This linguistic diversity has weakened cohesion among the Africa region countries with minimal involvement of French and Portuguese-speaking countries in WONCA activities. This calls for deliberate efforts to scale up WONCA membership to strengthen the voice of family doctors in advocacy and working together to develop quality primary care for better health and well-being of African populations.

## World Organization of Family Doctors membership

World Organization of Family Doctors offers different types of membership: full membership for national organisations that are representatives of general practitioners or family physicians within a country; associate membership for organisations that collaborate with WONCA and share its mission; academic membership for academic departments or training programmes of general practice or family medicine; individual membership for individuals interested in family medicine or general practice and honorary direct membership that is awarded to individuals for their contribution to WONCA and family medicine.^1^ Currently, the Africa region has only 12 full member organisations, a handful of direct members and only one academic member with no membership from French and Portuguese-speaking countries. This challenge of low membership is largely because of:

- A lack of training programmes in some countries and therefore no presence of family physicians. In such countries, if family physicians are present, they are very few who are trained in foreign countries. This explains the lack of awareness or recognition of the value of the specialty by governments, often seen as something for private practitioners. In most countries, family physicians are unavailable in human resources for health planning and very few or no posts exist.
- Difficulties with navigating the regulatory requirements to register the specialist professional associations with country governments, which is a pre-requisite to become a WONCA member organisation. Professional associations need their country’s legal status recognition before they can be enrolled as full WONCA member organisations.
- Very low numbers of family physicians with limited capacity to form a specialist professional association with register as a member organisation
- Low pay for family doctors causing inability to enrol as a direct member.
- Limited knowledge of the benefits of being a WONCA member among potential members. Such benefits include discounted conference registration fees, opportunities for collaboration and networking among others.

## What are we doing?

To scale up WONCA membership, the following actions have been performed:

- Establishment and provision of support to the Young Doctors network in the Africa region. The Young Doctors movement in Africa is known as AfriWon, which was established in 2013 at the World WONCA conference in Prague. WONCA Africa region supports AfriWon through mentorship and research collaborative programmes.
- Networking with other organisations and networks with similar vision and mission, for example, the Primary Care and Family Medicine (PRIMAFAMED) network for Africa.^2^ The PRIMAFAMED network is the official academic wing of WONCA Africa, providing research and educational capacity development workshops for family doctors and general practitioners in Africa.
- Establishment of a sub-committee of the executive on membership. This sub-committee supports countries to put in place specialist professional associations and applications to become WONCA full members.
- Distribution of the monthly WONCA news to different member organisations and individuals.
- Use of social media, particularly WhatsApp, to share WONCA information and activities.
- Supporting efforts to scale up training of family physicians within the region. For example, WONCA Africa through her member organisations based in different countries supported the establishment of the East Central and Southern Africa College of Family Physicians that will greatly contribute to the training of family doctors within the region.^3^

## Conclusions and way forward

World Organization of Family Doctors membership within the Africa region is still low, but the region has great potential, given the increasing numbers of family doctors being trained albeit at a slow pace. Networking and collaborating with other regional and national organisations that share the same mission as WONCA will greatly contribute to scaling up membership. The leaders of WONCA at regional and world level should continue supporting countries in training and capacity development of family doctors, as well as the application process. Deliberate support to French- and Portuguese-speaking countries will be necessary for all-inclusive scaling up of membership.

There is a need to address the awareness and understanding of family physicians in the region and how they can contribute to the model of care. WONCA Africa needs a budget to enable leadership in the region to more actively advocate for family medicine in different countries. Both, as a discipline to encourage countries to establish family medicine, and to scale up family medicine. The Memorandum of Understanding and potential collaboration between the World Health Organization and WONCA at a regional level could also be valuable.
